# Methylglyoxal-Derived Advanced Glycation Endproducts in Multiple Sclerosis

**DOI:** 10.3390/ijms18020421

**Published:** 2017-02-15

**Authors:** Suzan Wetzels, Kristiaan Wouters, Casper G. Schalkwijk, Tim Vanmierlo, Jerome J. A. Hendriks

**Affiliations:** 1 Department of Internal Medicine, Cardiovascular Research Institute Maastricht, Maastricht University, 6229 Maastricht, The Netherlands; suzan.wetzels@uhasselt.be (S.W.); kristiaan.wouters@maastrichtuniversity.nl (K.W.); c.schalkwijk@maastrichtuniversity.nl (C.G.S.); 2 Department of Immunology and Biochemistry, Biomedical Research Institute, Hasselt University, Martelarenlaan 42, 3500 Hasselt, Belgium; tim.vanmierlo@uhasselt.be

**Keywords:** multiple sclerosis, methylglyoxal, advanced glycation endproducts, glyoxalase system, receptor for advanced glycation endproducts

## Abstract

Multiple sclerosis (MS) is a demyelinating disease of the central nervous system (CNS). The activation of inflammatory cells is crucial for the development of MS and is shown to induce intracellular glycolytic metabolism in pro-inflammatory microglia and macrophages, as well as CNS-resident astrocytes. Advanced glycation endproducts (AGEs) are stable endproducts formed by a reaction of the dicarbonyl compounds methylglyoxal (MGO) and glyoxal (GO) with amino acids in proteins, during glycolysis. This suggests that, in MS, MGO-derived AGEs are formed in glycolysis-driven cells. MGO and MGO-derived AGEs can further activate inflammatory cells by binding to the receptor for advanced glycation endproducts (RAGE). Recent studies have revealed that AGEs are increased in the plasma and brain of MS patients. Therefore, AGEs might contribute to the inflammatory status in MS. Moreover, the main detoxification system of dicarbonyl compounds, the glyoxalase system, seems to be affected in MS patients, which may contribute to high MGO-derived AGE levels. Altogether, evidence is emerging for a contributing role of AGEs in the pathology of MS. In this review, we provide an overview of the current knowledge on the involvement of AGEs in MS.

## 1. Introduction

Multiple sclerosis (MS) is an inflammatory, demyelinating disease of the central nervous system (CNS) [1]. MS mainly manifests between the ages 20–40, affecting women twice as often as men [2]. The typical disease course, occurring in about 85% of MS patients, is relapsing-remitting (RR)-MS, in which there are episodes of acute neurological deficits (relapses) that result in disability, with full recovery between relapses [3]. Sixty-five percent of the RR-MS patients enter the secondary progressive stage of MS (SP-MS) within 5–15 years after the initial diagnosis [4]. The SP-MS phase is characterized by incomplete recovery between relapses and progression of the disease. Fifteen percent of MS patients show a progressive course from the onset of the disease, without relapses and remission. These patients are categorized as primary-progressive MS (PP-MS) patients. MS patients show a wide variety of symptoms, such as visual disturbance, paresthesia, ataxia, and muscle weakness, which originate from the damaged areas in the CNS [5].

MS is a complex disease. It is generally assumed that MS is triggered by environmental factors in genetically susceptible hosts. Family studies have revealed the genetic component in MS, and demonstrated a 20%–33% family recurrence rate and a 10–12 fold risk increase in first-degree relatives [6]. Several genes are associated with MS susceptibility, especially those encoding for the major histocompatibility complex (HLA DRB1*1501, HLA DQA1*0102, HLA DQB1*0602), which are responsible for 50% of the genetic risk for MS [7]. Genome-wide association studies (GWAS) have linked other immune-related genes to MS risk, including genes encoding the interleukin (IL)-17 receptor and IL-2 receptor, cytokines such as IL-12α and IL-12β, and genes associated with co-stimulatory molecules, including CD80, CD86, and CD37 [6]. In addition to genetic factors, there are also environmental factors that can contribute to the development of MS, such as active smoking [8], reduced levels of vitamin D [9], and infection with the Epstein–Barr virus [10], which are all confirmed risk factors for MS [11]. Reduced levels of vitamin D are linked with the geographic spread of MS, as these levels positively correlate with increasing latitude [12], due to reduced exposure to sunlight which is necessary for vitamin D production in the skin.

MS is an autoimmune disease of the CNS. The autoimmune response, which mainly involves autoreactive T-lymphocytes, macrophages, and CNS-resident microglia, is directed against CNS antigens [13]. Macrophages and microglia contribute to neuroinflammation and neurodegeneration by the secretion of pro-inflammatory mediators such as cytokines and chemokines, the degradation and phagocytosis of myelin, and the presentation of myelin antigens to autoreactive T-lymphocytes [13]. The interplay between the innate (e.g., macrophages and microglia) and the adaptive immune system at target locations is essential, as infiltrating T-lymphocytes require antigen presentation in order to be re-stimulated [14]. In addition to the cells of the immune system, astrocytes can also contribute to neuroinflammation since they exhibit functions that are similar to immune cells, such as the production of pro-inflammatory cytokines and chemokines [15].

It is clear that during MS, the CNS myelin is under attack by immune cells. There are two hypotheses for the role of immune cells in the development of lesions. First, a major hypothesis in MS pathology is that the immune activation of a specific CNS antigen occurs in the periphery and is then relocated to the CNS, the so-called “outside-in hypothesis” [16,17]. The activation of immune cells, mostly CD4^+^ T-lymphocytes, is thought be a result of molecular mimicry, in which cells are primed with a foreign antigen that resembles structures of autoantigens. The second, opposing, hypothesis states that an initiating event within the CNS, a primary infection or neuronal disturbances, causes the activation of resident microglia, and is called the “inside-out hypothesis” [16]. This immune reaction in the CNS leads to the recruitment of innate and adaptive immune cells from the periphery, which aggravates CNS inflammation. However, the exact cause of MS remains unknown.

## 2. Advanced Glycation Endproducts

Advanced glycation endproducts (AGEs) are increased in inflammatory diseases such as diabetes [18,19], atherosclerosis [19,20], obesity [21], and nonalcoholic steatohepatitis [22], but also in neuro-inflammatory diseases such as Alzheimer’s disease [23] and Parkinson’s disease [24]. Gaens et al. revealed that the AGE N^ε^-(carboxymethyl)-lysine (CML) is significantly increased in the liver [22] and visceral adipose tissue [21] of obese patients, when compared to controls, which was related to an increase in pro-inflammatory makers and thus inflammation. In Alzheimer’s disease, β-amyloid peptide depositions and neurofibrillary tangles are affected by glycation [25,26]. Moreover, Dalfó et al. have shown that glycation is present in the cerebral cortex, amygdala, and substantia nigra of healthy subjects, and that these are increased in Parkinson’s disease patients [24]. Also, AGEs are increased in the plasma and brain of MS patients [27,28]. The accumulation of AGEs in the plasma and CNS of MS patients, may contribute to neuroinflammation and the progression of MS.

### 2.1. Formation of AGEs

AGEs are stable endproducts of a non-enzymatic glycation reaction. The formation of AGEs (the Maillard-reaction) starts with the reaction of sugar aldehydes with the N-terminus of free-amino groups of proteins, to form a so-called Schiff base [29]. Rearrangements of the instable Schiff base lead to the formation of Amadori products. A small subset of Amadori products will undergo further irreversible reactions, leading to the formation of AGEs [29,30]. Frequently formed AGEs are N^ε^-(carboxymethyl)-lysine (CML), N^ε^-(carboxyethyl)-lysine (CEL), and pentosidine. The formation of AGEs via the Maillard-reaction is a slow process, taking weeks. In addition to the slow reaction, it is becoming clear that the majority of AGEs in vivo are mainly formed in a fast reaction of dicarbonyl compounds, such as methylglyoxal (MGO) and glyoxal (GO), with proteins [29].

### 2.2. Formation and Detoxification of Methylglyoxal

**Figure 1:**
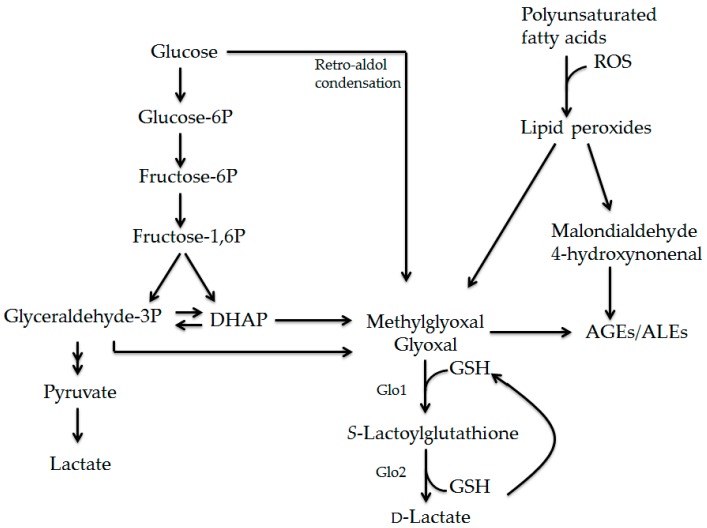
Formation of reactive dicarbonyl compounds and AGEs/ALEs via glucose and lipid intermediates. During glycolysis, glucose is converted into pyruvate and subsequently into lactate. Fragmentation of glyceraldehyde-3-phosphate (GAP) and dihydroxyacetone phosphate (DHAP) leads to the formation of methylglyoxal and glyoxal. In addition to glycolysis, lipid peroxidation of polyunsaturated fatty acids leads to the formation of lipid peroxides that can undergo fragmentation resulting in the formation of malondialdehyde, 4-hydroxynonenal, methylglyoxal, and glyoxal. Moreover, glyoxal can be directly created from glucose, via a retro-aldol condensation reaction. Incubation of these highly reactive compounds with proteins, lipids, and nucleic acids, leads to the fast formation of advanced glycation endproducts (AGEs) and advanced lipoxidation endproducts (ALEs). Methylglyoxal and glyoxal are detoxified via the glyoxalase system. First, methylglyoxal and glyoxal are converted to *S*-Lactoylglutathione by Glo1, which uses glutathione as a cofactor. Subsequently, *S*-Lactoylglutathione is metabolized to d-lactate by Glo-2. Glutathione gets recycled during this last step in the process.

MGO is produced as a byproduct of glycolysis, via the fragmentation of triosephosphates glyceraldehyde-3-phosphate (GAP) and dihydroxyacetone phosphate (DHAP), as shown in Figure 1 [31,32]. In addition, glyoxal can be directly created from glucose, via a retro-aldol condensation reaction, and indirectly, via GAP [33]. Moreover, reactive dicarbonyl compounds can also be formed as a result of lipid peroxidation, creating so-called advanced lipoxidation endproducts (ALEs). The lipid peroxidation of polyunsaturated fatty acids occurs under circumstances with increased oxidative stress and high amounts of reactive oxygen species (ROS). This leads to the formation of lipid peroxides. Lipid peroxides undergo fragmentation to produce reactive carbonyl compounds, such as malondialdehyde (MDA) and 4-hydroxynonenal (HNE), but also the dicarbonyl compounds, MGO and GO (Figure 1) [34].

Since there is a great variety in the free-amino groups in proteins, lipids, and nucleic acids, AGEs and ALEs represent a diverse and very large group of modifications. The interaction of MGO with arginine leads to the formation of the specific AGEs, methylglyoxal-derived hydroimidazolone 1 (MG-H1) and tetrahydropyrimidine (THP) [35]. In addition, MGO and GO can react with lysine to form CEL and CML, respectively. Since MGO and GO are formed during glycolysis and lipid peroxidation, CML and CEL can be regarded as both AGEs and ALEs [29].

The intracellular accumulation of the reactive carbonyls MDA and HNE, and dicarbonyl compounds MGO and GO, are highly toxic, because these compounds are potent glycating agents [31]. To reduce the toxic effects of reactive (di)carbonyl compounds and the formation of AGEs/ALEs, the body has several defense systems, such as glyoxalase, aldose reductase, aldehyde dehydrogenase, and carbonyl reductase pathways [31,33]. The glyoxalase system is the main defense system for reducing the toxicity of reactive dicarbonyl compounds. MGO, and to a lesser extent, GO, is detoxified by the glyoxalase system, a ubiquitous enzymatic pathway present in cytoplasm [32]. There are two enzymes responsible for the detoxification: glyoxalase-1 (Glo-1) and glyoxalase-2 (Glo-2). First, MGO is converted to *S*-Lactoylglutathione by Glo1, which uses glutathione (GSH) as a cofactor (Figure 1). Subsequently, *S*-Lactoylglutathione is metabolized to d-lactate by Glo-2. GSH gets recycled during this last step in the process, making it available for the new detoxification of MGO. The conversion of MGO by Glo1 is important because this is the rate-limiting step, and *S*-Lactoylglutathione is not as toxic to cells as MGO.

### 2.3. Biological Effects of Methylglyoxal and Advanced Glycation Endproducts

MGO can have several direct effects. MGO increases oxidative stress by inducing superoxide (O_2_^−^), hydrogen peroxide (H_2_O_2_), and peroxynitrite (ONOO^−^), but also by decreasing antioxidants and their mechanisms [36]. Moreover, cultured neuronal cells upregulate IL-1β expression and secretion after MGO stimulation [37], thereby contributing to inflammation. MGO is also able to induce apoptosis by increasing the Bax/Bcl-2 ratio and activation of caspase-9 and caspase-3, promoting the mitochondrial apoptosis pathway [38]. In addition to these direct effects, MGO is a potent glycating agent, resulting in the formation of AGEs which have biological effects through three general mechanisms. First, protein function can be altered by the intracellular glycation of proteins, resulting in distorted cell function [39]. Second is the modification of extracellular matrix proteins by AGEs, leading to altered interactions between the cells and proteins [29,40]. The third mechanism is the binding of AGEs to a variety of cell surface receptors, leading to the activation of downstream signaling pathways. The most described receptor is the multi-ligand receptor for advanced glycation end-products (RAGE). This receptor not only binds AGEs, but also amyloid proteins, high-mobility group B (HMGB), Mac-1, and S100 proteins [41,42], and is thought to be expressed on a variety of cell types involved in MS, such as monocytes/macrophages, T-lymphocytes, astrocytes, and endothelial cells. The binding of ligand to RAGE leads to increased intracellular oxidative stress and the activation of NF-κB, which increases the production of pro-inflammatory cytokines like IL-1α, IL-6, and TNFα [29,30]. However, there are more receptors which are known to bind AGEs, such as AGER1 [30,41], which is also expressed on monocytes/macrophages, T-lymphocytes, endothelial cells, and smooth muscle cells. AGER1 is a type I transmembrane protein that is supposed to facilitate AGE turnover by mediating the uptake, degradation, and removal of AGEs [40]. Moreover, AGER1 activation reduces the effects of RAGE signaling by the deacetylation of NF-κB via sirtuin-1 [40]. Therefore, AGER1 contributes to an anti-inflammatory status, as its signaling pathway leads to a decrease in oxidative stress and pro-inflammatory cytokines.

## 3. Advanced Glycation Endproducts in Multiple Sclerosis

There are several studies that have shown differences in AGE levels in MS patients, compared to controls. Moreover, there is evidence that AGEs contribute to the disease progression in MS. In the next section, we will summarize the literature describing AGE levels, the effects of AGEs, the glyoxalase system, the role of glycolysis, lipid peroxidation, and the receptor for advanced glycation end-products in MS.

### 3.1. Alterations in Advanced Glycation Endproduct Levels in Multiple Sclerosis

Previous research has demonstrated that AGEs are increased in the plasma and brain of MS patients [27,28]. Sternberg et al. investigated the diagnostic potential of plasma AGEs, specifically CML and CEL, in MS patients and healthy controls. The results showed that CEL plasma levels, but not CML levels, are higher in MS patients, compared to healthy controls [27]. Disease modifying treatments (DMTs) reduced CEL plasma concentrations. Furthermore, the presence of CML and RAGE was determined in paraffin-embedded brain sections of four relatively young MS patients [28]. It was found that CML and RAGE are expressed in astrocytes and macrophages both within, and in close proximity to, MS lesions. These studies have shown that AGEs are present in the brain and plasma of MS patients; however, it would be interesting to quantify the AGEs levels in the brains of MS patients and compare these levels to controls, to determine whether the increase of AGEs seen in the plasma of MS patients also reflects the AGE levels in the CNS, as this is the site where AGEs can activate their target cells.

### 3.2. The Effects of Advanced Glycation Endproducts on Key Cells in MS Development

**Figure 2:**
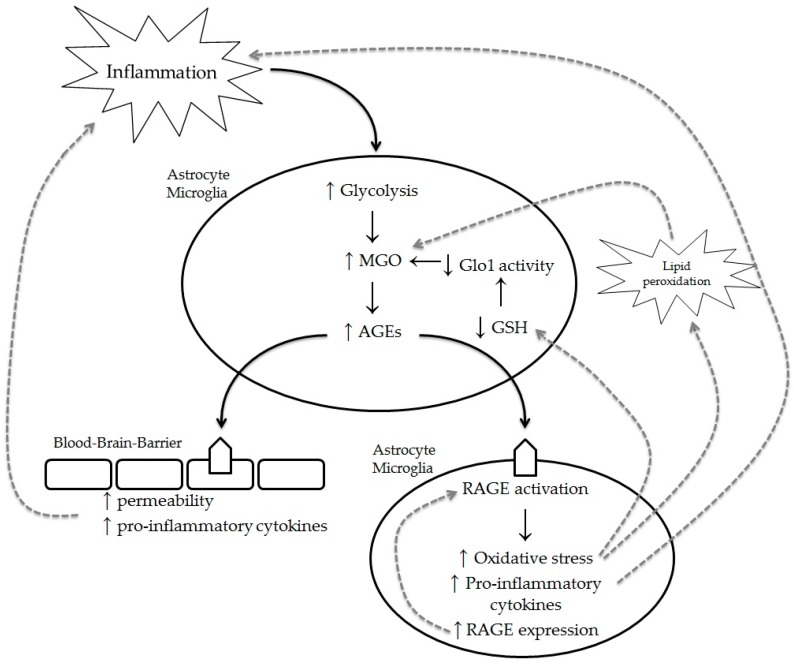
Schematic overview of the effects of methylglyoxal (MGO) on key cells in MS development. The inflammatory environment in the central nervous system (CNS) during MS leads to an increase (↑) in glycolysis in astrocytes and microglia. This induces (↑) the production of MGO and subsequently, AGEs. AGEs activate RAGE, which is present on astrocytes, microglia, and endothelial cells, leading to increased oxidative stress, the production of pro-inflammatory cytokines, and increased RAGE expression. Moreover, the BBB is affected by AGEs, leading to a loss of tight-junction proteins and thereby increasing permeability. Several positive feedback loops (dashed lines) are possible to further stimulate the inflammatory environment and moreover, increase the AGE levels in the CNS. The upregulation of RAGE upon its activation leads to an increased pathway activation and thus, oxidative stress and pro-inflammatory cytokines. Moreover, the production of pro-inflammatory cytokines contributes to the inflammatory status of the CNS. In addition, oxidative stress depletes (↓) glutathione (GSH), leading to decreased (↓) Glo1 activity, and stimulates lipid peroxidation, all of which contribute to the production of MGO, among others. ↑ upward arrow indicates increased production, ↓ downwards arrow indicates decreased production and/or activity.

Methylglyoxal is a potent glycating agent, leading to increased levels of MGO-derived AGEs which can exert their effects via their receptor, RAGE. Key cells in MS development such as microglia, astrocytes, and endothelial cells (in the BBB), express RAGE, making them targets for AGEs. It can be hypothesized that MGO-derived AGEs act as accelerators of MS lesion pathology, and function as a detrimental positive feedback loop, as illustrated in Figure 2. It has been reported that activation of microglia by AGEs leads to an increased expression and secretion of pro-inflammatory cytokines, such as TNFα, IL-1β, and IL-6 [43,44,45]. Moreover, stimulation with AGEs leads to increased levels of RAGE [45,46], creating a positive feedback loop that promotes inflammation. In addition to microglia, astrocytes are abundantly present in the CNS and also express RAGE, making them susceptible to AGE-RAGE activation. Indeed, it is reported that stimulation of astrocytes with glucose-modified bovine serum albumin, which can be regarded as AGEs, leads to increased TNFα and IL-6 secretion [47]. Furthermore, a glucose-rich environment, which is present in the CNS of MS patients, induces a pro-inflammatory phenotype in astrocytes which contributes to neuroinflammation.

The blood–brain barrier (BBB) is required to maintain homeostasis within the CNS and block the entry of toxic stimuli, infectious agents, and peripheral immune cells. The BBB consists of endothelial cells that are attached to each other by tight junctions. These tight junctions, comprised of different tight junction proteins such as occludins and claudins, restrict the passive influx of molecules and cells into the CNS [48]. Moreover, besides endothelial cells, astrocytes and pericytes are support the BBB. Endothelial cells of the BBB are affected when stimulated with AGEs, leading to the loss of tight junction protein expression and thus, increasing the permeability of the BBB [49,50]. In addition, endothelial cells secrete pro-inflammatory cytokines that contribute to inflammation. Glycation of the underlying matrix proteins was shown to lead to increased BBB permeability [49]. AGE-activated astrocytes increase the production of vascular endothelial growth factor, and decrease the production of glial cell line-derived neurotrophic factor, also leading to an increase in BBB permeability [51]. Considering all of these results, we can hypothesize that AGEs act as accelerators of MS lesion pathology, by inducing a pro-inflammatory phenotype in microglia and astrocytes. This also leads to increased RAGE expression, which can act as a positive feedback loop by inducing more pro-inflammatory mediators. In addition, AGEs disrupt BBB function which leads to the increased infiltration of peripheral immune cells into the CNS, contributing to neuroinflammation and neurodegeneration.

### 3.3. The Glyoxalase System in Multiple Sclerosis

The major precursor in the formation of AGEs, MGO, and to a lesser extent, GO, can be detoxified by the glyoxalase system. As mentioned before, this system uses GSH as a cofactor, which is reused in the glyoxalase system, as d-lactate is formed. In the CNS, the level of GSH is maintained by active intracellular GSH synthesis, originating from astrocytes, but also from neurons [52]. In addition to the de novo synthesis, GSH can be recycled by glutathione reductase, which converts the oxidized form of glutathione (GSSH) into the reduced form (GSH). In 2002, Calabrese et al. determined the amount of GSH in cerebrospinal fluid (CSF) samples of MS patients with the NADPH-dependent GSSG reductase method, revealing significantly decreased GSH in the CSF of MS patients [53]. Moreover, Choi et al. developed a method to non-invasively measure GSH in vivo, using MRI, and found that GSH in the fronto-parietal area of the brain was significantly decreased in SPMS patients, compared to controls [54,55]. The decrease in GSH concentration in MS patients may limit the detoxification of MGO by the glyoxalase system, leading to the accumulation of MGO in the cells, ultimately leading to an increase in MGO-derived AGEs. In addition to GSH availability, Sidoti et al. determined the frequency of the A111E polymorphism present in the *Glo-1* gene, as this particular polymorphism is known to have a decreased detoxification capacity [56]. The A111E polymorphism leads to the change of alanine into glutamic acid (Ala111Glu) in protein sequence. The frequency of the EE genotype, which has the glutamic acid on both chromosomes, was significantly increased in RR-MS patients compared to controls (59.8% vs. 49.3%, *p* < 0.0001) [57], suggesting that decreased Glo-1 activity can contribute to increased MGO-derived AGE-levels in MS patients, compared to controls. 

### 3.4. Increased Glycolysis as an Underlying Mechanism for the Formation of Methylglyoxal-Derived Advanced Glycation Endproducts in Multiple Sclerosis

The formation of AGEs via reactive dicarbonyl compounds mainly occurs in highly metabolic active cells which rely on glycolysis, such as macrophages [58], microglia [59], and astrocytes [60,61,62]. In 1962, Karnovsky reported that phagocytosis leads to increased glycolysis in macrophages [63]. This demonstrates that, in MS, glycolysis is increased in phagocytes after the uptake of myelin. Supporting this, Bogie et al. used the micro-array analysis of myelin-treated macrophages to reveal that genes involved in glycolysis are induced [64], which likely results in the formation of AGEs in myelin-containing macrophages.

Glucose is the main energy source of the brain, where the energy requirements are high [65]. Nijland et al. investigated the distribution of specific glucose transporters in brain tissue of MS patients and non-neurological controls, and found that glucose transporter 1 (GLUT1) and 4 (GLUT4) are increased in MS lesions [66]. GLUT1 is expressed in the brain microvasculature, which ensures the transport of glucose over the BBB and uptake of glucose by astrocytes [67]. GLUT4 is expressed on astrocytes and endothelial cells. It is known that demyelinated axons require more energy to maintain proper conduction of signals [68]. Therefore, an upregulation of nutrient transporters within MS lesions and increased glycolysis is necessary. Indeed, previous studies have revealed that MS patients have an increased glucose and lactate metabolism within lesions in the CNS, which was observed with positron emission tomography and magnetic resonance spectroscopy [69,70]. The energy needed for signaling processes such as postsynaptic and action potentials, is mainly derived from astrocytes, featured by a high glycolytic rate [60,61,62]. In addition to astrocytes, oligodendrocytes also appear to be glycolytic, since the glycolytic activity is higher in white matter, which consists of high numbers of oligodendrocytes compared to grey matter [71]. Funfschilling et al. proposed a hypothetical model in which glucose is used for ATP generation and serves the synthesis of myelin lipids at the onset of myelination [72]. Moreover, it is also suggested that in post-myelinated oligodendrocytes, glycolysis is used to maintain survival. These data indicate that, in MS, not only the astrocytes, but also the oligodendrocytes, are potential sources of glycolysis-derived reactive dicarbonyl compounds, and thus of AGEs.

### 3.5. Increased Lipid Peroxidation as an Underlying Mechanism for the Formation of Methylglyoxal-Derived Advanced Glycation Endproducts in Multiple Sclerosis

In addition to the glycolysis-derived formation of AGEs, AGEs are also formed during lipid peroxidation via the formation of reactive carbonyls MDA and HNE, and dicarbonyl compounds, such as MGO and GO. The formation of lipid-derived AGEs is initiated by reactive oxygen species (ROS) (Figure 1) [34]. ROS are highly reactive small molecules that have an unpaired electron, and which have the ability to give rise to new free radicals [73]. ROS production can be rapidly increased due to oxidative phosphorylation in mitochondria, phagocytosis, and in enzymatic reactions which catalyze oxidases [74]. Under physiological conditions, concentrations of ROS remain low, as a result of anti-oxidative mechanisms which include enzymatic reactions (superoxide dismutase and catalase) and non-enzymatic molecules (vitamin C, vitamin E, GSH). However, the CNS is sensitive to oxidative stress and the production of ROS, due to the high rate of oxygen utilization and a relatively poor anti-oxidant defense system [75]. In addition, immune cells are a great source of ROS. During MS, activated microglia and infiltrated monocyte-derived macrophages accumulate in the CNS. Both microglia and macrophages produce large quantities of ROS [76]. A recent study from Guan et al. showed that MS patients have increased levels of the lipid peroxidation marker 8-*iso*-PGF2α in their urine, when compared to healthy controls, indicating that lipid peroxidation is increased [77]. Moreover, the levels of urinary 8-*iso*-PGF2α corresponded with MS disease severity. Since the CNS is rich in polyunsaturated fatty acids, an increased amount of lipid peroxides can be formed due to lipid peroxidation. Van Horssen et al. compared the oxidative damage in MS lesions to normal appearing white matter (NAWM) and healthy controls [78]. Data from this study revealed that oxidative damage to proteins, nucleotides, and lipids, is increased in MS lesions, compared to NAWM and controls. Furthermore, this oxidative damage was mostly found in hypertrophic astrocytes and phagocytic macrophages in active demyelinated lesions [78]. Moreover, Wang et al. revealed that MDA, a reactive carbonyl compound which is able to induce ALEs, is elevated in RR-MS patients [79]. The results from the above studies show that oxidative stress and lipid peroxidation are increased in MS patients. This may lead to an increased MGO, and subsequently, increased AGE production in MS patients.

### 3.6. Receptors for Advanced Glycation Endproducts in Multiple Sclerosis

RAGE is expressed on various cell types that are involved in MS. Andersson et al. determined that RAGE was upregulated in active MS lesions and in CNS lesionsm in experimental autoimmune encephalomyelitis (EAE); an animal model of MS [80]. In 2003, Yan et al. examined the role of RAGE during EAE development and in MS [81]. It was shown that RAGE immunoreactivity is increased in brain samples from MS patients, especially in mononuclear phagocytes and CD4^+^ T cells. This was confirmed in the spinal cord tissue of EAE mice. There is also experimental evidence that RAGE contributes to the disease progression of MS. Treatment of EAE mice with sRAGE, the cleaved variant of RAGE which prevents activation of membrane-bound RAGE [82], or specific RAGE blocking of antibodies which partially protects them from developing EAE, suggested that the activation of RAGE by ligands is necessary for the development of EAE. In contrast, Liliensiek et al. found that full body RAGE deficiency (RAGE^-/-^) did not affect EAE development [83]. However, cell specific overexpression of RAGE on hematopoietic and endothelial cells, led to a significant increase in EAE severity, compared to wild type controls. This suggests that RAGE expression on immune and endothelial cells is involved in the perpetuation of, but not in the initiation of, neuroinflammation [83]. These data, showing no protective effect of full body RAGE deficiency during EAE development, are in contrast with the data of Yan et al, who revealed that treatment with sRAGE partially protects mice from EAE development. There are multiple explanations as to why these studies show contrasting results. One could speculate that there is a difference in the peripheral effects of RAGE, which are mainly blocked by sRAGE, compared to the full body of RAGE deficiency. Moreover, there may be a difference in the cell types affected by RAGE deficiency, and in the treatment with sRAGE- or RAGE-blocking antibodies. Therefore, more experimental research needs to be conducted, to obtain conclusive results about the role of RAGE during EAE and neuro-inflammatory responses.

Interestingly, Sternberg et al. showed that the percentage of RAGE-positive monocytes and T-lymphocytes was significantly increased in MS patients [84]. While membrane-bound RAGE was increased, sRAGE was decreased in MS patients and inversely related to the disability of the patient, indicating that the receptor is involved in MS progression and can be used as a biomarker [85]. The increase of RAGE-positive monocytes and T-lymphocytes in MS patients can lead to a more pro-inflammatory phenotype of these cells. In addition, sRAGE has therapeutic potential, as it prevents the activation of RAGE, which is necessary for EAE development.

Several polymorphisms for RAGE have been described, including −429 T/C, −407 to 345 deletion, −374 T/A, +20 T/A, and a substitution of Glycine with Serine at amino acid 82 (G82S) [86,87]. In 2009, Tiszlavicz et al. found that the −374 T/A polymorphism was different between the MS patients and healthy controls in a Hungarian population, leading to a higher frequency of the T/T genotype in MS patients, meaning that both chromosomes contain the nucleotide Thymine [88]. Although the frequency of the G82S polymorphism was not significantly different in Tiszlavicz’s Hungarian population, Li et al. showed that the odds ratio of the G82S polymorphism is significantly different in a Chinese study cohort comparing MS patients with healthy controls, with a higher frequency of 82S in MS patients [89]. Although these two studies revealed differences in RAGE polymorphisms in MS patients compared to controls, GWAS could not confirm these polymorphisms in large cohorts. These results indicate that these two polymorphisms are likely dependent on ethnic background, or that interaction with different environmental factors might contribute to the difference seen in the populations.

In addition to RAGE, more receptors that are able to bind AGEs, are of interest. One of these receptors is AGER1. We can only speculate about the function of AGER1 in MS. This AGE receptor ameliorates the negative effect of the AGE-RAGE axis by suppressing NF-κB activity [90], and thereby reduces the production of pro-inflammatory cytokines. The expression of AGER1 can be influenced by the AGE burden in the microenvironment, as extensive, prolonged AGE exposure down-regulates the expression of AGER1 [40]. AGER1 might be a promising target in MS that can decrease the AGE load within the CNS, stimulating an anti-inflammatory environment. Suppression of NF-κB not only decreases the production of pro-inflammatory cytokines, but also leads to an increased phagocytosis potency of macrophages [91]. Phagocytosis of myelin debris by macrophages is essential for inducing the remyelination of axons [92]. Therefore, AGER1 activation may be beneficial for remyelination and may prevent neuronal damage. However, no studies have yet investigated the contribution of AGER1 to MS pathology.

## 4. Conclusions and Future Prospective

AGEs, especially CEL and CML, are increased in the plasma and brain of MS patients [27,28]. Several studies found increased AGE levels in the CNS of MS patients, and there is plenty of evidence that glycolysis and lipid peroxidation are increased in MS. This potentially leads to high MGO-derived AGE levels in the plasma and CNS of these patients. Moreover, a number of studies have revealed that the expression of the receptor RAGE, and the major detoxification enzyme of MGO, Glo1, are altered during MS. Altogether, emerging evidence suggests a contributing role of the MGO and AGE-RAGE axis in the disease progression of MS. However, the exact role of the AGE-RAGE axis and its main detoxification enzyme Glo1, in the progression of MS, needs to be elucidated. First, the AGE content in the CNS of MS patients and controls, but also in the commonly used animal model of MS, the EAE mouse model, needs to be determined. Second, the specific cell types involved in AGE accumulation need to be identified, and the mechanisms by which AGEs modulate MS disease pathology have to be elucidated. Finally, the mouse model of MS should be used to investigate whether possible treatment strategies that reduce AGEs or block the AGE-RAGE axis, result in a better disease outcome. One possible therapeutic is pyridoxamine, which scavenges dicarbonyl compounds and prevents the formation of AGEs. The prevention of tissue inflammation and damage, possibly by the inactivation of AGEs or downstream mechanisms, is of great importance for the development of new and improved treatments for MS.

## Abbreviations

AGER1	Advanced Glycation Endproduct Receptor 1
AGEs	Advanced Glycation Endproducts
ALEs	Advanced Lipoxidation Endproducts
BBB	Blood–brain barrier
CD	Cluster of Differentiation
CEL	N^ε^-(carboxyethyl)-lysine
CML	N^ε^-(carboxymethyl)-lysine
CNS	Central Nervous System
CSF	Cerebrospinal Fluid
DHAP	Dihydroxyacetone Phosphate
DMTs	Disease Modifying Treatments
EAE	Experimental Autoimmune Encephalomyelitis
EDSS	Expended Disability Status Score
GAP	Glyceraldehyde-3-phosphate
Glo-1	Glyoxalase-1
Glo-2	Glyoxalase-2
GLUT	Glucose Transporter
GO	Glyoxal
GSH	Glutathione
GWAS	Genome-wide Association Studies
HLA	Human Leukocyte Antigen
HMGB	High-Mobility Group B
HNE	4-Hydroxynonenal
IL	Interleukin
MDA	Malondialdehyde
MG-H1	Methylglyoxal-derived Hydroimidazolone 1
MGO	Methylglyoxal
MS	Multiple Sclerosis
NADPH	Nicotinamide Adenine Dinucleotide Phosphate
NAWM	Normal Appearing White Matter
NF-κB	Nuclear Factor-κB
PBMCs	Peripheral Blood Mononuclear Cells
PP-MS	Primary Progressive Multiple Sclerosis
RAGE	Receptor for Advanced Glycation Endproducts
ROS	Reactive Oxygen Species
RR-MS	Relapsing Remitting Multiple Sclerosis
SP-MS	Secondary Progressive Multiple Sclerosis
sRAGE	Soluble Receptor of Advanced Glycation Endproducts
THP	Tetrahydropyrimidine
TNFα	Tumor Necrosis Factor alpha
